# Sphingosine 1-Phosphate- and C-C Chemokine Receptor 2-Dependent Activation of CD4^+^ Plasmacytoid Dendritic Cells in the Bone Marrow Contributes to Signs of Sepsis-Induced Immunosuppression

**DOI:** 10.3389/fimmu.2017.01622

**Published:** 2017-11-23

**Authors:** Anna Smirnov, Stephanie Pohlmann, Melanie Nehring, Shafaqat Ali, Ritu Mann-Nüttel, Stefanie Scheu, Anne-Charlotte Antoni, Wiebke Hansen, Manuela Büettner, Miriam J. Gardiasch, Astrid M. Westendorf, Florian Wirsdörfer, Eva Pastille, Marcel Dudda, Stefanie B. Flohé

**Affiliations:** ^1^Department of Orthopedics and Trauma Surgery, University Hospital Essen, University of Duisburg-Essen, Essen, Germany; ^2^Institute of Medical Microbiology and Hospital Hygiene, University of Düsseldorf, Düsseldorf, Germany; ^3^Cells in Motion, Cluster of Excellence, University of Münster, Münster, Germany; ^4^Institute of Medical Microbiology, University Hospital Essen, University of Duisburg-Essen, Essen, Germany; ^5^Institute of Functional and Applied Anatomy, Hannover Medical School, Hannover, Germany; ^6^Medical Faculty, Institute of Cell Biology (Cancer Research), University of Duisburg-Essen, Essen, Germany

**Keywords:** dendritic cells, bone marrow, differentiation, polymicrobial sepsis, immunosuppression, monocytes, sphingosine-1 phosphate, chemokines

## Abstract

Sepsis is the dysregulated response of the host to systemic, mostly bacterial infection, and is associated with an enhanced susceptibility to life-threatening opportunistic infections. During polymicrobial sepsis, dendritic cells (DCs) secrete enhanced levels of interleukin (IL) 10 due to an altered differentiation in the bone marrow and contribute to the development of immunosuppression. We investigated the origin of the altered DC differentiation using murine cecal ligation and puncture (CLP), a model for human polymicrobial sepsis. Bone marrow cells (BMC) were isolated after sham or CLP operation, the cellular composition was analyzed, and bone marrow-derived DCs (BMDCs) were generated *in vitro*. From 24 h on after CLP, BMC gave rise to BMDC that released enhanced levels of IL-10. In parallel, a population of CD11c^hi^MHCII^+^CD4^+^ DCs expanded in the bone marrow in a MyD88-dependent manner. Prior depletion of the CD11c^hi^MHCII^+^CD4^+^ DCs from BMC *in vitro* reversed the increased IL-10 secretion of subsequently differentiating BMDC. The expansion of the CD11c^hi^MHCII^+^CD4^+^ DC population in the bone marrow after CLP required the function of sphingosine 1-phosphate receptors and C-C chemokine receptor (CCR) 2, the receptor for C-C chemokine ligand (CCL) 2, but was not associated with monocyte mobilization. CD11c^hi^MHCII^+^CD4^+^ DCs were identified as plasmacytoid DCs (pDCs) that had acquired an activated phenotype according to their increased expression of MHC class II and CD86. A redistribution of CD4^+^ pDCs from MHC class II^−^ to MHC class II^+^ cells concomitant with enhanced expression of CD11c finally led to the rise in the number of CD11c^hi^MHCII^+^CD4^+^ DCs. Enhanced levels of CCL2 were found in the bone marrow of septic mice and the inhibition of CCR2 dampened the expression of CD86 on CD4^+^ pDCs after CLP *in vitro*. Depletion of pDCs reversed the bias of splenic DCs toward increased IL-10 synthesis after CLP *in vivo*. Thus, during polymicrobial sepsis, CD4^+^ pDCs are activated in the bone marrow and induce functional reprogramming of differentiating BMDC toward an immunosuppressive phenotype.

## Introduction

Dendritic cells (DCs) are professional antigen-presenting cells and are present in lymphoid and non-lymphoid tissues where they serve as “sentinels” of the immune system. Diverse subsets of DCs exist: conventional DCs characteristically express high levels of CD11c along with major histocompatibility complex (MHC) class II and co-stimulatory molecules that are strongly increased during bacterial infection (1). Moreover, upon contact with diverse microbial components, DCs secrete interleukin (IL) 12 and other pro-inflammatory cytokines and subsequently become very potent in the induction of T helper (Th) type 1 responses and in the activation of natural killer (NK) cells (2, 3). In turn, Th1 and NK cells secrete interferon (IFN) γ that increases the bactericidal activity of phagocytes and thereby supports the elimination of the pathogens (4). Due to their capacity to orchestrate the collaboration of immune cells, DCs play a decisive role in the defense against numerous bacterial infections.

Plasmacytoid DCs (pDCs) express intermediate and low levels of CD11c and MHC class II, respectively, along with B220, bone marrow stromal cell antigen (Bst) 2, and Ly6C but are negative for the myeloid marker CD11b (5, 6). The hallmark of pDCs is their ability to release extraordinarily high amounts of type I interferons that renders pDCs indispensable for the defense against viral infections (7). Upon stimulation, pDCs increase their expression of MHC class II and co-stimulatory molecules, may release diverse cytokines and chemokines, and become potent antigen-presenting cells (8, 9).

In the steady-state, conventional DCs differentiate from common DC progenitors (CDP) in the bone marrow to pre-DCs that leave the bone marrow and settle in peripheral tissues where they finally differentiate to conventional DCs (10). During inflammation, DCs may additionally develop from bone marrow-derived Ly6C^hi^ inflammatory monocytes and acquire features similar to conventional DCs including high expression of MHC class II and costimulatory molecules along with Th cell priming (11, 12). pDCs may also originate from CDP but unlike conventional DCs, they finally differentiate in the bone marrow before they are released into the circulation (13). However, the intermediate stages of pDC differentiation as well as a recently discovered heterogeneity within the circulating pDCs population are a matter of current scientific debate (14, 15).

The migration of leukocytes is tightly regulated by diverse chemotactic molecules. Thereby, C-C chemokine ligand (CCL) 2 is of major importance as it interrupts the retention of monocytes and other immune cells in the bone marrow upon binding to its receptor C-C chemokine receptor (CCR) 2 and subsequently induces their mobilization (16–18). Moreover, CCL2 is released at the site of inflammation and attracts circulating leukocytes through CCR2 (19).

Sphingosine 1-phosphate (S1P) is a lipid mediator and is present in plasma and lymph at high concentrations (20). Leukocytes express diverse receptors for S1P and migrate toward enhanced S1P concentrations from the tissue into the blood or lymph vessels (20). FTY720 is an analog of S1P and serves as a functional antagonist of S1P as it triggers the internalization of S1P receptors and desensitizes the cell to S1P (21). Blocking of S1P receptors through FTY720 leads to the retention of T lymphocytes in lymphoid organs and to the depletion of these cells from blood (22).

Sepsis is the dysregulated host response to generalized mostly bacterial infections and still represents the major cause of death on intensive care units (23). Sepsis is characterized by an overwhelming systemic inflammation due to the massive release of pro-inflammatory cytokines, activation of the complement system, and leukocyte recruitment that may cause multi-organ failure (24). Despite modern intensive care medicine and diverse therapeutic approaches that aimed to limit hyperinflammation, the mortality of patients with septic shock is still high (25). Parallel to pro-inflammatory mediators, anti-inflammatory cytokines, such as IL-10 and TGF-β, are released and cells of the innate and adaptive immune system become disabled to eradicate the pathogens (26). After surviving early hyperinflammation, the immunosuppressive state dominates and strongly increases the risk for life-threatening nosocomial infections (27). The pathomechanisms that cause sepsis-associated immunosuppression are still incompletely understood.

During polymicrobial sepsis DCs rapidly lose their ability to respond to microbial components with the secretion of IL-12 (28). This impaired responsiveness of DCs is maintained for several weeks (29–31). Instead, they predominantly release IL-10 (28, 29, 31). Consequently, DCs from septic mice are impaired in Th1 and cytotoxic T cell priming (29, 32). We have previously shown that during polymicrobial sepsis, DCs undergo functional reprogramming during their differentiation in the bone marrow (29). *De novo* generated DCs secrete high levels of IL-10 that interferes with Th1 priming, they inhibit the function of NK cells, and mediate enhanced susceptibility to secondary infection (29). We shortly defined these DCs as “dysfunctional DCs.” While the ontogeny of DCs has been extensively studied in the past, little information exists on the mechanisms that are responsible for the functional programming of DCs during differentiation. Thus, here, we aimed to investigate the origin of the functional reprogramming of DCs from bone marrow during murine polymicrobial sepsis.

## Materials and Methods

### Animals

Female wild-type BALB/c mice (6–8 weeks old, 17–21 g) were obtained from ENVIGO, Rossdorf, Germany or from Janvier Labs, Saint Berthevin Cedex, France. Myeloid differentiation factor (MyD) 88^−/−^ (33), toll-like receptor (TLR) 4^−/−^ (34), and recombination-activating gene (RAG) 2^−/−^ (35) mice on BALB/c background were bred at the local animal facility of the University Hospital Essen. All mice were kept under specific pathogen-free conditions and had access to standard rodent food and water *ad libitum*. All animal experiments were performed according to the ethical principles and guidelines for scientific experiments. The protocol has been approved by the local ethic committee Landesamt für Natur-, Umwelt-, und Verbraucherschutz (LANUV), North-Rhine-Westphalia.

### Induction of Polymicrobial Sepsis and Applications

Polymicrobial sepsis was induced through cecal ligation and puncture (CLP) as described previously (29). Briefly, after anesthesia with an intramuscular injection of ketamine (100 mg/kg) and xylazine (10 mg/kg), the mice underwent a midline laparotomy and the cecum was exposed and ligated by 50%. The cecum was punctured once with a 27-gauge needle and approximately 1 µl of cecum content was extruded. Thereafter, the cecum was replaced, 1 ml sterile saline was administered intraperitoneally (i.p.) for resuscitation, and the peritoneum was closed in two layers. CLP caused a moderate sepsis with a mortality rate of 20% within the first 2 days. Where indicated mice received the CCR2 antagonist RS102895 (5 mg/kg body weight Tocris Biosciences, Bio-Techne, Avonmouth, Bristol, UK) dissolved in DMSO i.p. 6 h before sham or CLP operation and every 6 h thereafter until the end of the experiment. This dosage maintains a constant concentration of circulating RS102895 (36). Where indicated, the functional S1P receptor antagonist FTY720 (2 mg/kg body weight; Fingolimod, LC Labs, Woburn, MA, USA) dissolved in 20% 2-hydroxypropyl-β-cyclodextrin (2-HPCD) was intravenously (i.v.) applied immediately before sham and CLP operation and 12 h thereafter. Control mice received the solvents only. In some experiments, the mice were treated i.p. with antibodies against PDCA-1/Bst2 (clone JF05-1C2.4.1, Miltenyi Biotech; 100 µg/mouse) or with the respective rat IgG2b isotype control antibodies 6 h before and 24 h after surgery.

### Splenectomy

Splenectomy was performed 3–4 weeks before sham or CLP operation. Therefore, mice were anesthetized with an intramuscular injection of ketamine (100 mg/kg) and xylazine (10 mg/kg). After a 1 cm incision of the left lateral part of the peritoneum, the spleen was exposed and removed after ligation of the blood vessels. After resuscitation with 1 ml sterile saline i.p., the peritoneum was closed in two layers. Control mice underwent laparotomy only. For analgesia, carprofen (4 mg/kg body weight Paracarp, Norbrook Laboratories, Corby, Great-Britain) was injected subcutaneously immediately after surgery and 24 h thereafter.

### Preparation and Culture of Cells and Lavage

VLE RPMI 1640 (Biochrom, Berlin, Germany) supplemented with 10% fetal calf serum (FCS, Biochrom), 10 mM HEPES (Biochrom), 2 mM glutamine, 0.06 mg/ml penicillin, 0.02 mg/ml gentamicin, and 0.05 mM 2-Mercapthoethanol (all from Sigma-Aldrich, Taufkirchen, Germany) was used as culture medium (CM).

Whole blood was collected by cardiac puncture and transferred into EDTA tubes. For erythrocyte depletion, 200 µl of blood were lysed using 2 ml Red Blood Cell Lysing Buffer (Sigma-Aldrich).

To collect the peritoneal lavage and cells, 3 ml PBS were injected into the peritoneal cavity, aspirated, and centrifuged. The supernatant was harvested as peritoneal lavage. Thereafter, 4 ml of CM containing 5 IU/ml Heparin Ratiopharm (Ratiopharm GmbH, Ulm, Germany) were injected into the peritoneal cavity, aspirated, and pooled with the cells previously obtained from the lavage with PBS. Care was taken to avoid the injury of blood vessel.

For the isolation of total spleen cells, the spleen was removed and digested with collagenase as described previously (28). Erythrocytes were lysed with Red Blood Cell Lysing Buffer (Sigma-Aldrich). Splenic DCs were isolated using magnetic beads coupled with antibodies against CD11c (MagniSort CD11c Positive Selection Kit, Invitrogen, Carlsbad, CA, USA) as recommended by the manufacturer and were cultured as described (28).

Bone marrow cells (BMCs) were flushed out of femurs and tibiae with CM, gently dissociated, and filtered through a 30 µm filter. To generate bone marrow-derived dendritic cells (BMDCs), BMCs (2 × 10^5^/ml) were cultured in CM containing 20 ng/ml recombinant murine granulocyte/macrophage-colony-stimulating factor (GM-CSF; R&D Systems, Wiesbaden, Germany) for 7–9 days as described previously (37). Non-adherent cells were harvested and BMDC were cultured in the absence or presence of 5 µg/ml CpG (ODN1668, InvivoGen, Toulouse, France) for 18 h. The supernatant was stored at −20°C for further analyses. Alternatively, BMCs were cultured in the presence of Flt3L (R&D Systems) for 7 days in order to generate conventional and pDCs as described (38).

For the preparation of bone marrow lavage, femurs and tibiae were flushed with 2 ml PBS. After centrifugation, the supernatant was harvested and stored at −20°C for further analyses.

### Flow Cytometry

For surface staining, cells were incubated with diverse combinations of fluorochrome-labeled antibodies against CD11c (clone N418), MHC class II (clone M5/114.15.2), CD4 (clone RM4-5), Ly6C (clone AL-21), CD103 (clone 2E7), CD11b (clone M1/70), Bst2 (clone eBio927), B220 (clone RA3-6B2), SIRP-α (clone P84), CCR9 (clone eBioCW1.2), or Siglec-H (clone eBio440c) for 15 min in the dark at 4°C as indicated. Finally, the cells were washed with Cell Wash (BD Biosciences). Appropriate isotype controls were used to define the threshold of negative versus positive staining. All antibodies were purchased from BD Biosciences or from eBioscience (Frankfurt am Main, Germany). Data were acquired using a FACSCalibur or FACSCanto II (BD Biosciences). Data analysis was performed using CellQuest Pro, FACSDiva Software (both BD Biosciences), or NovoExpress (ACEA Biosciences, San Diego, CA, USA).

### Cell Sorting

Bone marrow cells were adjusted to a density of 1 × 10^7^ cells/ml in PBS supplemented with 2% FCS and were stained with fluorescent antibodies against MHC class II, CD4, and CD11c as described above. The cells were washed twice with PBS and CD11c^hi^ MHCII^+^CD4^+^ cells were sorted using a FACS Aria (BD Biosciences). The positive fraction was used for cytospin preparations (see below) and for RNA extraction, the negative fraction was used as depleted BMC for cell culture. Flt3L generated DCs were sorted as CD3^−^CD19^−^CD11c^+^CD11b^+^B220^−^ conventional DCs and CD3^−^CD19^−^CD11c^+^CD11b^−^B220^+^ pDCs.

### Cytospin and Giemsa Staining

Twenty thousand cells were mounted onto a glass slide using a CytospinTM 4 (Thermo Fisher Scientific, Dreieich, Germany) for 5 min at 400 rpm and were fixed with ice-cold methanol–acetone (1:1 vol/vol). Cells were stained with Giemsa-solution for 5 min.

### Quantification of Proteins

The content of IL-10, IL-12p70, and CCL2 was quantified using ELISA (all DuoSet, R&D Systems) or cytometric bead array (CBA, BD Biosciences) as recommended by the manufacturers.

### Real-time PCR

RNA was extracted using the RNeasy micro kit (Qiagen, Hilden, Germany) and reverse transcribed using the Sensiscript Reverse Transcriptase (Qiagen) or SuperScript III Reverse Transcriptase (ThermoFisher Scientific, Waltham, MA, USA). Real-time PCR was performed with the following primers: *e2-2* (fwd TGGGCTCAGGGTACGGAACT, rev CAGAGCCACGCCATCTTCAC), *id2* (fwd GACAGAACCAGGCGTCCAGG, rev AGCTCAGAAGGGAATTCAGATG), *il-10* (fwd CTGGACAACATACTGCTAACCGACTC, rev ATTTCTGGGCCATGCTTCTCTGC), β*-actin* (fwd CGCTCAGGAGGAGCAATG, rev TGACAGGATGCAGAAGGAGA), *rps9* (fwd CTGGACGAGGGCAAGATGAAGC, rev TGACGTTGGCGGATGAGCACA). Wobble primers for several *ifn-*α subtypes were: fwd ATGGCTAGRCTCTGTGCTTTCCT and rev AGGGCTCTCCAGAYTTCTGCTCTG. PCR was run on the iQ5 iCycler (Bio-Rad) or on the CFX96 RealTime C1000 Thermo Cycler (Bio-Rad) using FastStar Taq Man Probe Master (Roche) and Mesa Green qPCR Mastermix Plus SYBR Assay with fluorescein (Thermo Fisher Scientific), respectively according to the manufacturers’ recommendations. The target gene expression was normalized on the housekeeping genes β*-actin* or *rps9* and was calculated as 2^−ΔCt^ with ΔCt = Ct target-Ct housekeeping.

### Statistical Analyses

Data are shown as individual values with median and interquartile range or as mean ± SD or SEM. Differences between two groups were tested using Mann–Whitney *U*-test. Multiple comparisons were performed with the Kruskal–Wallis test followed by Dunn’s posttest or by One-way ANOVA followed by Newman–Keuls Multiple Comparison test. Normalized values were tested using the One-sample *t*-test. A *p*-value <0.05 was considered as significant. Statistical analyses and the preparation of graphs were performed using GraphPad Prism 5.0.

## Results

### CD11c^hi^MHCII^+^CD4^+^ Cells Accumulate in the Bone Marrow after CLP and Modulate the Differentiation of BMDC

**Figure 1:**
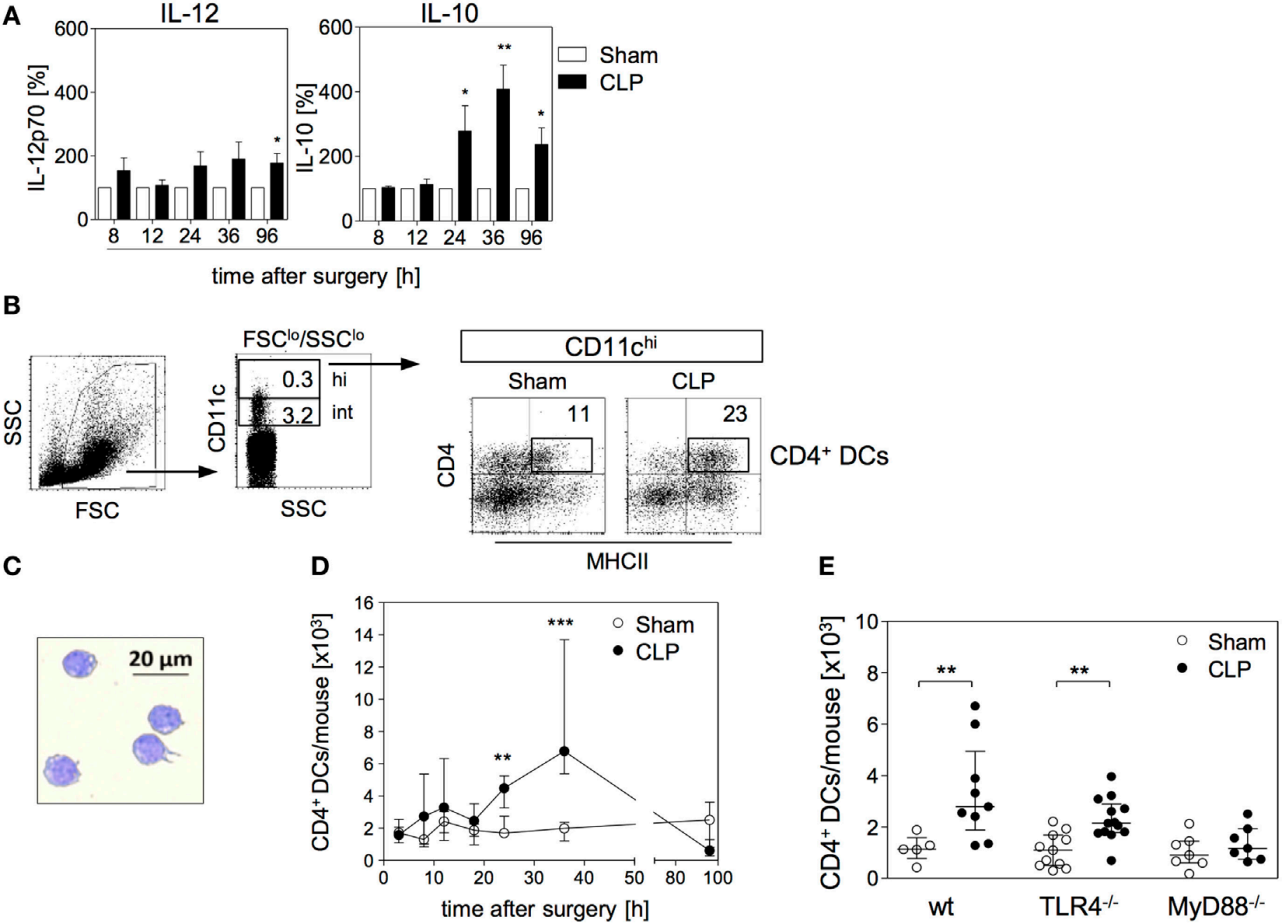
Functional reprogramming of BMDC during sepsis is associated with the expansion of a distinct population of CD4^+^ dendritic cells (DCs) in the bone marrow. At different time points after sham operation or cecal ligation and puncture (CLP), bone marrow cells (BMCs) were isolated. **(A)** BMDCs were generated from pooled BMC from *n* = 3–4 mice per group and were stimulated with CpG. The release of IL-12 (left) and IL-10 (middle) into the supernatant was quantified. For each experiment, the cytokine production of BMDC from CLP mice was normalized to the respective values from BMDC of sham mice (set as 100%). The bars indicate the mean + SEM of 3–10 experiments per time point. Statistical differences were tested using the one-sample *t*-test. In the absence of CpG, BMDC did not release detectable amounts of IL-12 or IL-10 (data not shown). **(B)** Gating strategy of “CD4^+^ DCs.” BMC were stained for CD11c, CD4, and major histocompatibility complex (MHC) class II. CD11c^hi^ cells were gated among SSC^lo^/FSC^lo^ cells. Dot plots show the expression of CD4 and MHC class II on gated CD11c^hi^ cells from sham and CLP mice of one representative experiment (36 h after surgery). Numbers indicate the percentage of gated cells or cells within the respective quadrant. **(C)** Giemsa staining of a representative Cytospin preparation of sorted CD4^+^ DCs from CLP mice. Bright field images were obtained at 20× magnification. **(D)** Absolute number of CD11c^hi^MHCII^+^CD4^+^ DCs [gated as shown in **(B)**] in the bone marrow per mouse 3–96 h after surgery. Data show the median/interquartile range of individual mice (*n* = 4–12 per group) for each time point. **(E)** Wildtype (wt), TLR4^−/−^, or MyD88^−/−^ mice underwent sham or CLP operation. After 36 h, BMCs were isolated and the number of CD4^+^ DCs was determined as shown in panel **(B)**. The scatter plot shows individual values from *n* = 5–13 mice per group. Horizontal lines indicate the median/interquartile range. Statistical differences between sham and CLP mice were tested using the Mann–Whitney *U*-test. **p < 0.01; ***p < 0.001. DCs, dendritic cells; BMDC, bone marrow-derived DCs.

We have previously shown that BMC from septic mice when cultured in the presence of GM-CSF *in vitro* give rise to BMDC that resemble splenic DCs during sepsis in terms of increased IL-10 synthesis (29). Bone marrow cell cultures in the presence of GM-CSF mimic the differentiation of DCs under inflammatory conditions. Due to their enhanced secretion of IL-10 in response to bacterial stimuli such as CpG immunostimulatory oligonucleotides BMDC from septic mice possess immunosuppressive properties (29). To elucidate the sepsis-induced changes in the bone marrow that may result in the altered differentiation of BMDC, we investigated the CpG-induced cytokine secretion pattern of BMDC generated from bone marrow at different time points after CLP. To adjust inter-assay variations, the values obtained from BMDC of septic mice were normalized to the values received from BMDC of sham mice (set as 100%; a representative data set of absolute values is given in Figure S1 in Supplementary Material). At least up to 12 h after CLP, the bone marrow gave rise to BMDC that secreted moderately enhanced levels of IL-12 but similar amounts of IL-10 in comparison with bone marrow from sham mice. From 24 h after CLP, BMDC displayed a strong increase in IL-10 production (Figure 1A).

We next searched for changes in the cellular composition of the bone marrow that were associated with the altered differentiation of BMDC 24 h and later after sepsis induction. We identified an enlarged population of cells that highly expressed CD11c, along with MHC class II and CD4 by 36 h after CLP (Figure 1B). Strong expression of CD11c together with MHC class II is characteristic for terminally differentiated DCs. Morphologically, the CD11c^hi^MHCII^+^CD4^+^ cells displayed a large nucleus and partly showed the characteristic dendritic cell shape (Figure 1C). Therefore, we termed the CD11c^hi^MHCII^+^CD4^+^ cells “CD4^+^ DCs” hereafter. Few CD4^+^ DCs were also present in the bone marrow of sham mice (Figure 1D). The number of CD4^+^ DCs in the bone marrow rose 24 h after CLP and this population further expanded at least until 36 h (Figure 1D) and, thus, paralleled the increased release of IL-10 from BMDC generated from bone marrow of septic mice. By 96 h after CLP, the population of CD4^+^ DCs had largely disappeared from the bone marrow (Figure 1D). The rise in the number of CD4^+^ DCs in the bone marrow after CLP was dependent on MyD88 but not on TLR4 (Figure 1E).

**Figure 2:**
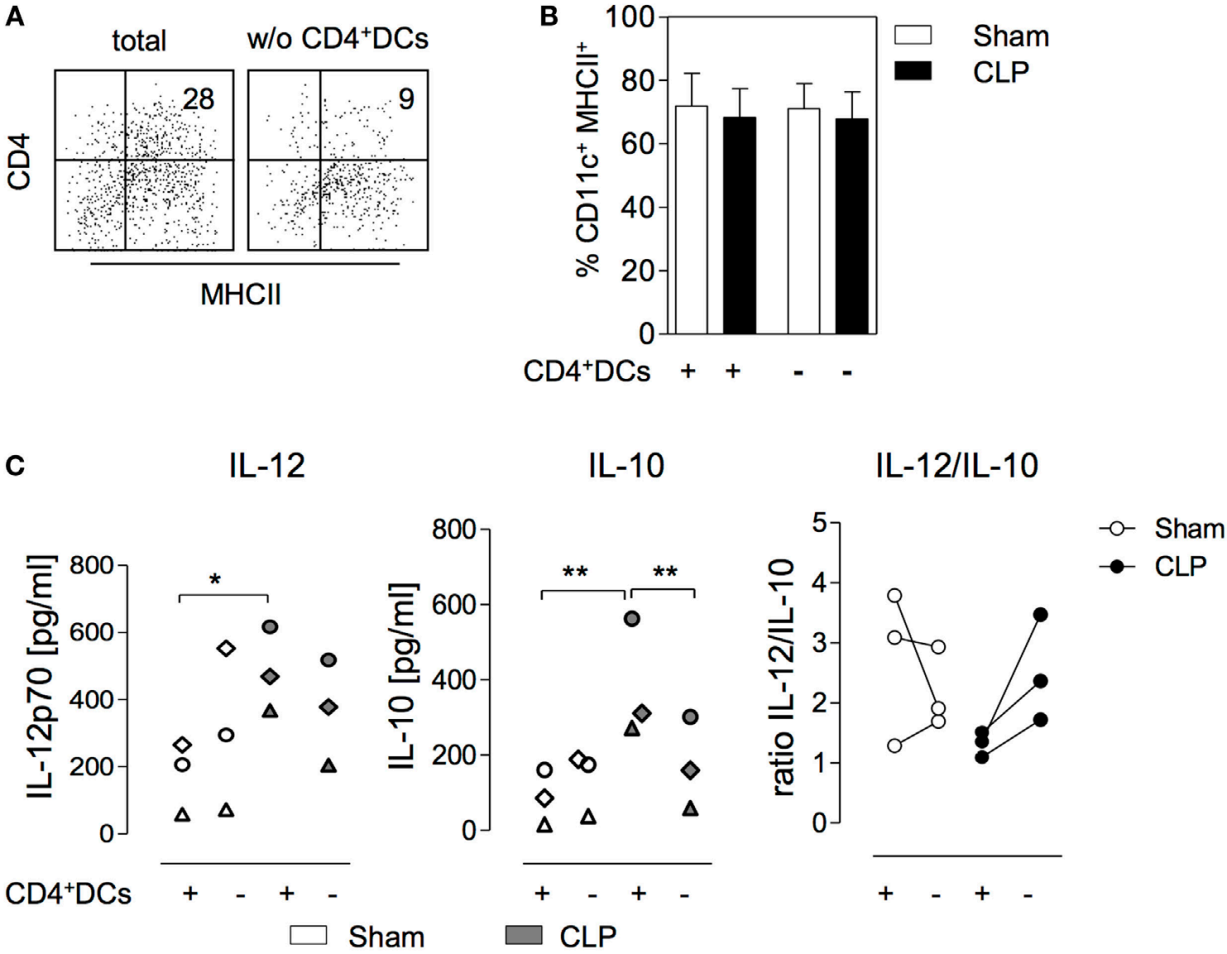
CD11c^hi^MHCII^+^CD4^+^ dendritic cells (DCs) within the bone marrow favor the differentiation of IL-10-secreting BMDC after cecal ligation and puncture (CLP). Bone marrow cells (BMC) were isolated 36 h after sham or CLP operation, were pooled per group, and stained for CD11c, MHC class II, and CD4. Complete BMC (+) or cells that were depleted of CD11c^hi^MHCII^+^CD4^+^ DCs by cell sorting (−) were used to generated BMDC *in vitro*. **(A)** Representative dot plots indicating the expression of CD4 and MHC class II on gated CD11c^hi^ DCs in the bone marrow of CLP mice before (total) and after (w/o) depletion of CD4^+^ DCs. **(B)** Frequency of CD11c^+^ MHCII^+^ BMDC generated from complete or depleted BMC from sham and CLP mice *in vitro*. Data show mean + SD from three independent experiments each with *n* = 2–4 mice per group. **(C)** BMDC cultures were set up in triplicates, were stimulated with CpG, and the release of IL-12 (left) and IL-10 (middle) was determined. Data show the mean values of the triplicate cultures for separate experiments (*n* = 3; each with 2–4 mice per group). The experiments are distinguished by individual symbols. Statistical differences were tested using one-way ANOVA followed by Newman–Keuls Multiple Comparison test. The quotient of the mean concentrations of IL-12 and IL-10 is expressed as “ratio IL-12/IL-10” (right). *p < 0.05; **p < 0.01. DCs, dendritic cells; BMDC, bone marrow-derived DCs.

In order to address the potential impact of CD4^+^ DC in the bone marrow on the differentiation of BMDCs, CD4^+^ DCs were depleted from BMC (through cell sorting) obtained 36 h after surgery. Total BMC and cells after depletion of CD4^+^ DCs were used to set up cultures for the generation of BMDC (Figure 2A). At the end of the *in vitro* culture, the percentage of CD11c^+^MHC class II^+^ BMDC did not differ irrespective of the origin of the bone marrow (sham or CLP) or of the presence of CD4^+^ DCs (Figure 2B). With regard to the cytokine secretion, prior depletion of CD4^+^ DCs from BMC of sham mice did not significantly change the CpG-induced release of IL-12 and IL-10 from BMDC (Figure 2C). Likewise, the absence of CD4^+^ DCs in the bone marrow of septic mice did not affect the secretion of IL-12 from generated BMDC (Figure 2C). In contrast, BMDC released significantly less IL-10 when they had been generated from BMC of CLP mice that had been depleted from CD4^+^ DCs before the onset of the culture (Figure 2C). Consequently these BMDC displayed an increased IL-12/IL-10 ratio in comparison to BMDC that differentiated from total bone marrow of CLP mice (Figure 2C). Thus, CD4^+^ DCs accumulate in the bone marrow during sepsis and modulate the cytokine secretion pattern of subsequently differentiating BMDC toward increased IL-10 production.

### CD4^+^ DCs Expand in the Bone Marrow in a S1P-Dependent Manner

In general, the enlargement of a specific population may be caused by local cell proliferation or by the recruitment of cells from the periphery. The analysis of the proliferation marker Ki-67 indicated a comparable though low percentage of proliferating CD4^+^ DCs in the bone marrow of both sham and CLP mice (Figure S2 in Supplementary Material).

**Figure 3:**
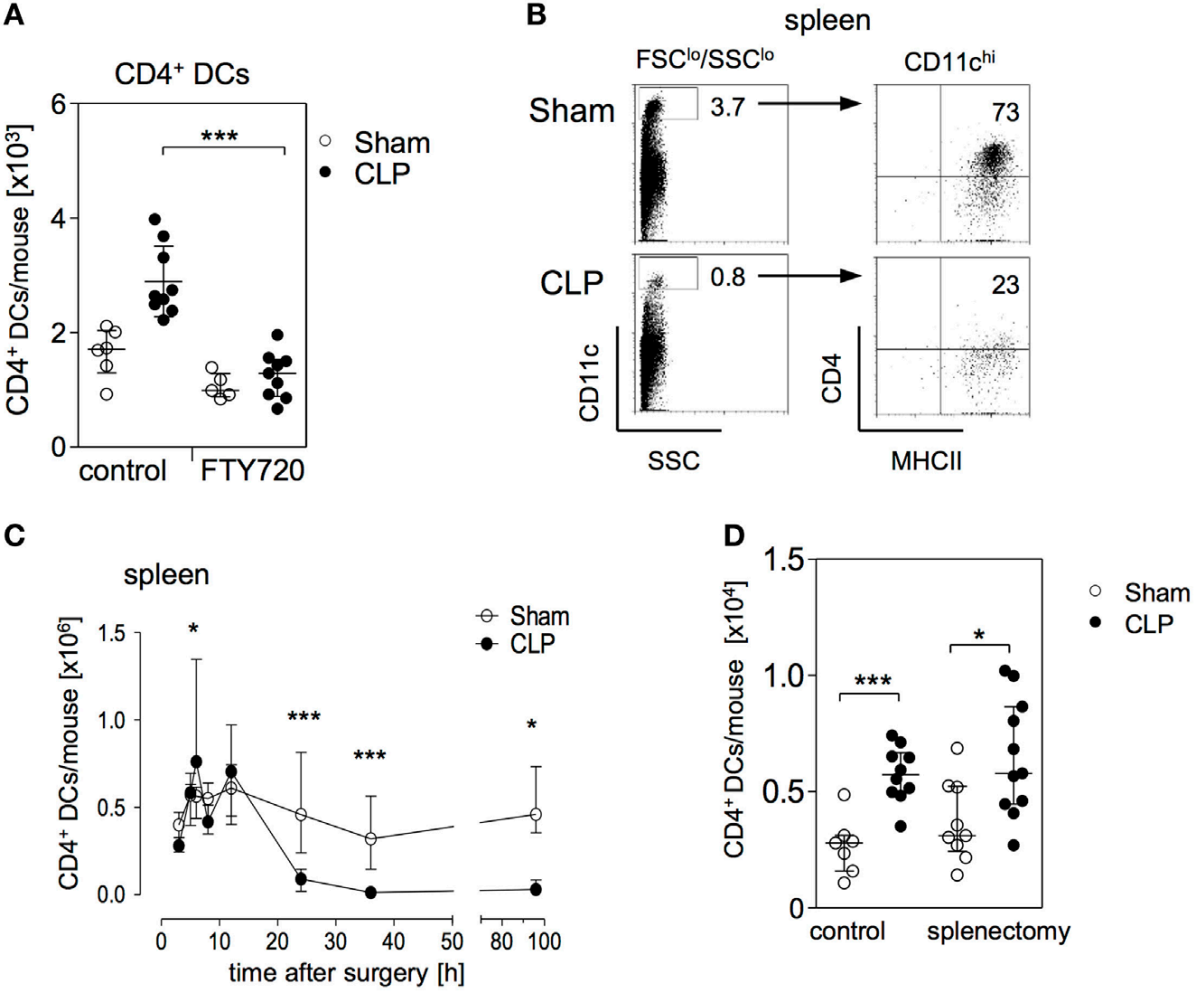
The expansion of the CD11c^hi^MHCII^+^CD4^+^ DC population in the bone marrow is mediated by S1P receptors and does not rely on splenic dendritic cells (DCs). **(A)** Mice were treated with the functional sphingosine 1-phosphate (S1P) receptor antagonist FTY720 or with the solvent as control before sham or cecal ligation and puncture (CLP) operation. After 36 h, the number of CD11c^hi^MHCII^+^CD4^+^ DC in the bone marrow was determined. The scatter plot shows the values of individual mice (*n* = 5–9 mice per group) and the median/interquartile range as horizontal lines. Statistical differences were tested using the Kruskal–Wallis test followed by Dunn’s posttest. **(B,C)** CD11c^hi^MHCII^+^CD4^+^ DCs in the spleen. The gating strategy **(B)** was equivalent to the gating of CD4^+^ DCs in the bone marrow. Numbers indicate the percentage of gated cells or cells within the respective quadrant. Representative dot plots of one sham and one CLP mouse (36 h after surgery) are shown. **(C)** At different time points after sham or CLP operation total spleen cells were isolated and the number of CD11c^hi^MHCII^+^CD4^+^ DCs was quantified (*n* = 4–21 per group). **(D)** Mice underwent splenectomy or laparotomy as control surgery 4 weeks before sham or CLP operation. After 36 h, the number of CD4^+^ DC in the bone marrow was quantified. The scatter plot shows the values of individual mice. Horizontal lines depict the median/interquartile range (*n* = 7–11 mice per group). Statistical differences between sham and CLP were tested using the Mann–Whitney *U*-test. *p < 0.05; ***p < 0.001. DCs, dendritic cells.

Sphingosine 1-phosphate contributes to the migration of DCs and lymphocytes through lymphatic and blood vessels, respectively (39, 40). Since the bone marrow is not connected to the lymphatic system the circulation is the only way to enter the bone marrow from the periphery. In order to evaluate whether S1P played a role in the accumulation of CD4^+^ DCs in the bone marrow after CLP, the mice were treated with FTY720 or with its solvent before CLP or sham operation. The sepsis-induced accumulation of CD4^+^ DCs in the bone marrow was completely blocked upon application of FTY720 (Figure 3A). In contrast, FTY720 did not affect the number of CD4^+^ DCs in the bone marrow of sham mice (Figure 3A).

The spleen is a large reservoir for conventional CD11c^hi^CD4^+^ DCs (gated as shown in Figure 3B). The expansion of CD4^+^ DCs in the bone marrow 24 and 36 h after CLP was paralleled by a striking decrease of CD4^+^ DCs in the spleen (Figures 3B,C). We further investigated whether the enlarged population of CD4^+^ DCs in the bone marrow was associated with the loss of DCs in the spleen during the course of sepsis. Therefore, mice underwent splenectomy or control laparotomy 4 weeks before sham or CLP operation. Subsequent analysis showed that prior splenectomy did not prevent the sepsis-induced accumulation of CD4^+^ DCs in the bone marrow (Figure 3D).

**Figure 4:**
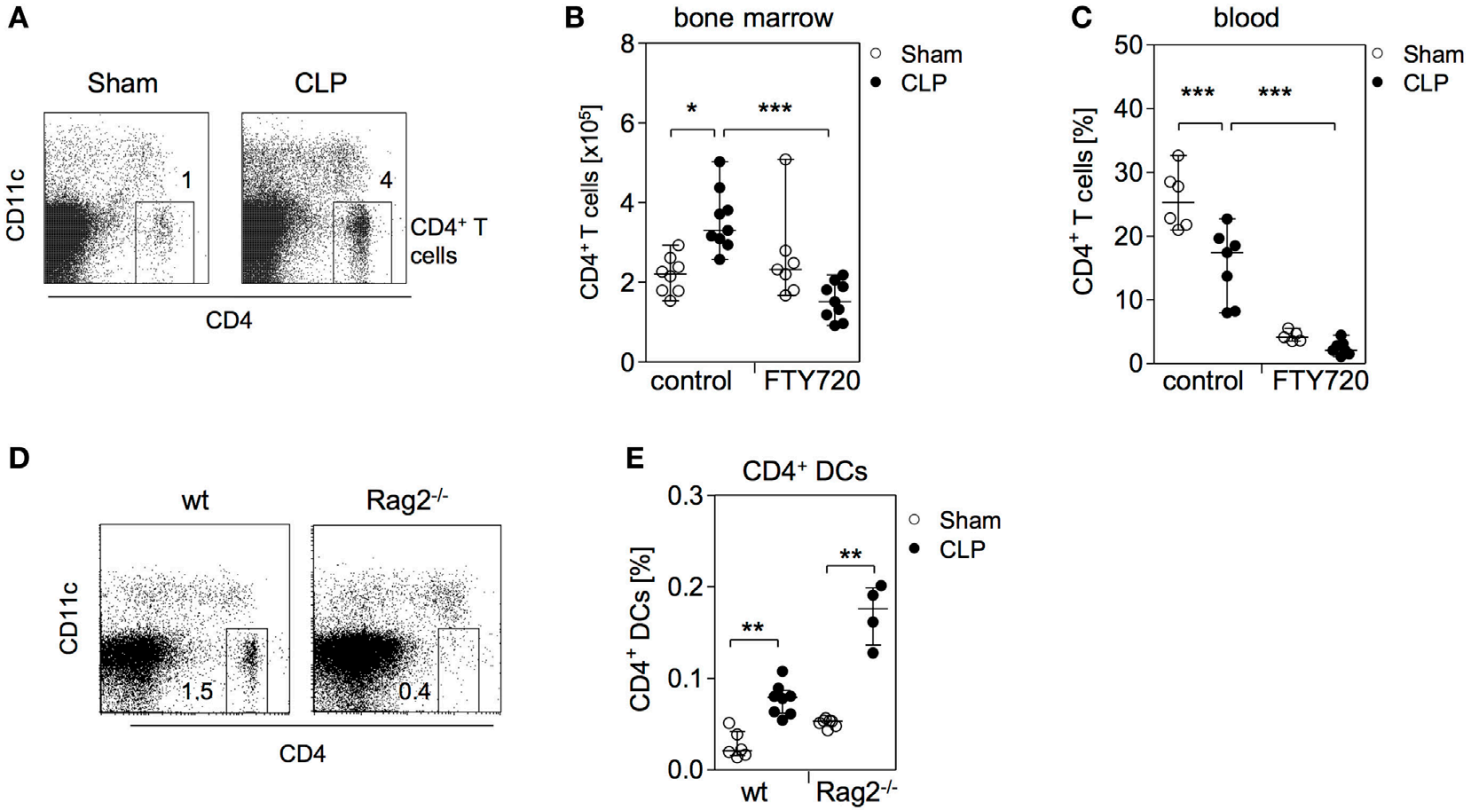
CD4^+^ T cells accumulate in parallel to CD4^+^ DCs in the bone marrow during sepsis. **(A)** Representative dot plots of bone marrow cells (BMC) from one sham and one cecal ligation and puncture (CLP) mouse 36 h after surgery. CD11c^−^CD4^+^ T lymphocytes were gated. **(B,C)** Mice were treated with the functional sphingosine 1-phosphate receptor antagonist FTY720 or with the solvent before sham or CLP operation. After 36 h, the number and frequency of CD4^+^ T cells in the bone marrow **(B)** and blood **(C)**, respectively, were determined. Data show the values of individual mice (*n* = 5–9 mice per group) with median/interquartile range. **(D)** Analysis of the bone marrow of one representative naive wildtype (wt) and one Rag2^−/−^ mouse for the presence of CD4^+^ T cells. Numbers indicate the percentage of gated cells. **(E)** Wildtype and Rag2^−/−^ mice underwent sham or CLP operation. After 36 h, BMC were isolated and the frequency of CD11c^hi^MHCII^+^CD4^+^ DCs was determined according to the gating strategy shown in Figure 1B. Data indicate the values of individual mice (*n* = 4–8 per group). Horizontal lines depict the median/interquartile range. Significant differences between sham and CLP were tested using the Mann–Whitney *U*-test. *p < 0.05; **p < 0.01; ***p < 0.001. DCs, dendritic cells.

We further examined the impact of FTY720 on the distribution of CD4^+^ T cells in the bone marrow during sepsis. Numerous studies have shown that lymphocytes undergo apoptosis-induced cell death in secondary lymphoid organs and in the blood within 24 h after induction of sepsis (41, 42). In contrast, there exists limited if not any information on the behavior of T lymphocytes in the bone marrow during sepsis. Interestingly, we observed an enlarged population of CD4^+^ T cells in the bone marrow by 36 h after CLP that was dependent on S1P receptor function (Figures 4A,B). In line with a previous study on the effect of pharmacologically induced modulation of S1P signaling during sepsis (43), the application of FTY720 caused severe lymphopenia in the blood of both groups (Figure 4C).

The comparable S1P-dependent expansion of CD4^+^ T cells and CD4^+^ DCs implied a relationship between these two cell types in the bone marrow. To examine whether the accumulation of CD4^+^ DCs in the bone marrow was linked to the increased number of CD4^+^ T cells, sepsis was induced in Rag2 knockout mice that lack CD4^+^ T cells (Figure 4D). The accumulation of CD4^+^ DCs was also observed in Rag2 deficient mice (Figure 4E). Thus, the expansion of the CD4^+^ DC population in the bone marrow after CLP is dependent on S1P but independent of CD4^+^ T cells that accumulate in parallel.

### The Rise in the CD4^+^ DC Number in the Bone Marrow Is Regulated by CCR2

**Figure 5:**
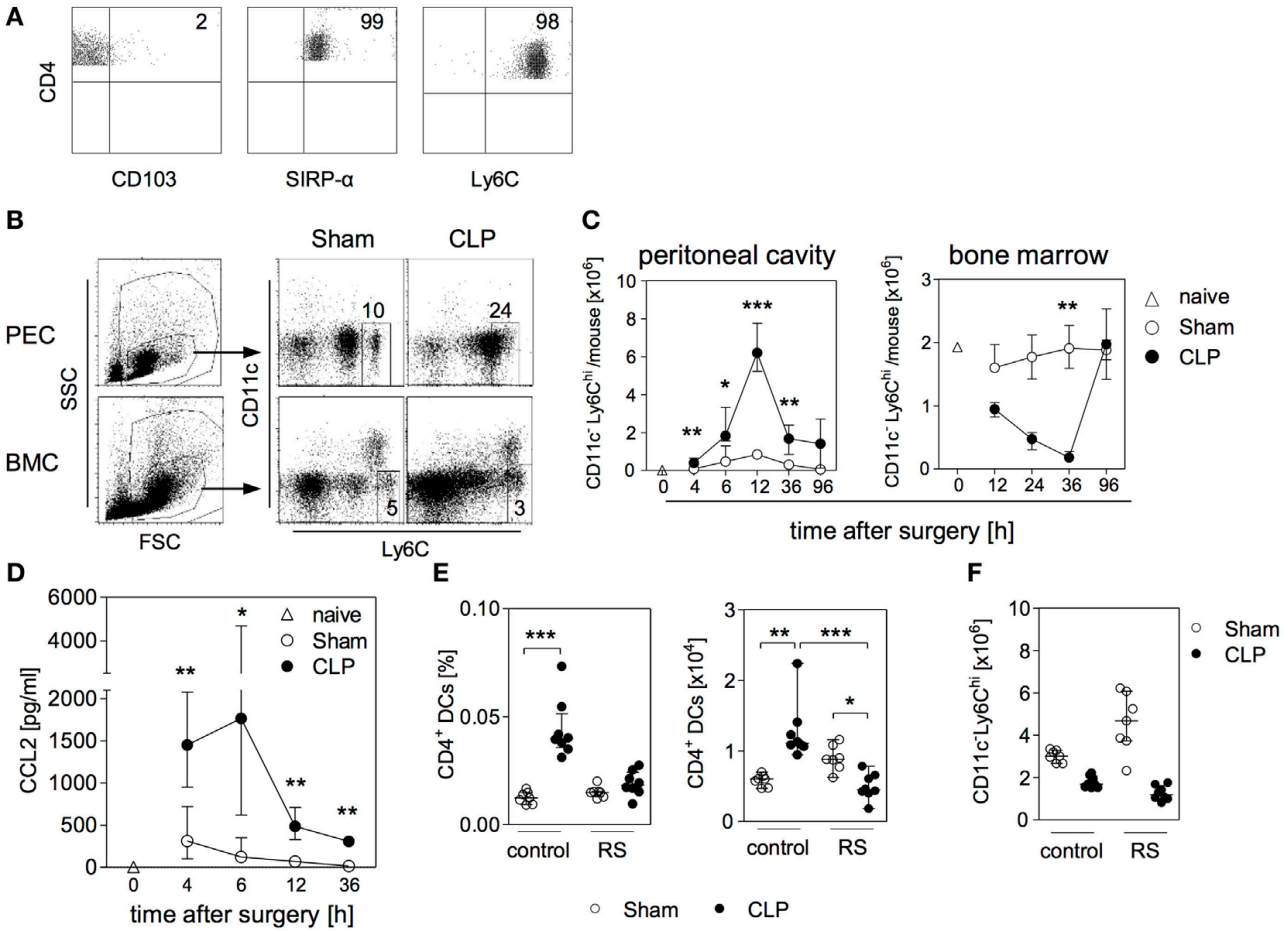
Relevance of C-C chemokine receptor (CCR) 2 in the expansion of CD4^+^ DCs in the bone marrow during sepsis. At different time points after sham or cecal ligation and puncture (CLP) operation, bone marrow cells (BMC), peritoneal exudate cells (PEC), and peritoneal lavage were isolated. **(A)** Expression of CD103, SIRP-α, and Ly6C on CD4^+^ DCs in the bone marrow of CLP mice (36 h after surgery) gated according to the strategy shown in Figure 1B. **(B)** Representative dot plots of PEC (12 h after surgery) and BCM (36 h after surgery) each from one sham and one CLP mouse. Monocytes were gated as CD11c^-^Ly6C^hi^. Numbers indicate the percentage of Ly6C^hi^ monocytes among total leukocytes **(C)** Absolute number of Ly6C^hi^ monocytes in the peritoneal cavity and in the bone marrow. Data show median/interquartile range of individual mice (*n* = 4–8 per group). **(D)** Content of CCL2 in the peritoneal lavage. Data show median/interquartile range of individual mice (*n* = 4–12 per group). The white triangles each indicate the respective value obtained from one naïve mouse. Statistical difference between sham and CLP mice was tested using Mann–Whitney *U*-test. **(E,F)** Mice were treated with the selective CCR2 antagonist RS102895 (RS) or with the solvent as control before and after surgery. 36 h after surgery, BMCs were isolated and the frequency and absolute number of CD11c^hi^MHCII^+^CD4^+^ DCs **(E)** as well as the number of Ly6C^hi^ monocytes **(F)** were quantified. Statistical differences were tested using the Kruskal–Wallis test with Dunn’s posttest. *p < 0.05; **p < 0.01; ***p < 0.001. DCs, dendritic cells.

We further analyzed the expanding CD4^+^ DC population in the bone marrow after CLP for the expression of CD103, SIRP-α, and Ly6C that are associated with conventional DC lineages and/or monocyte-derived DCs, respectively (12, 44–47). The CD4^+^ DCs were negative for CD103 but expressed SIRP-α and Ly6C suggesting a monocytic origin (Figure 5A). Despite the knowledge on the importance of monocytes in inflammatory diseases, only few data exist on their behavior during CLP-induced polymicrobial sepsis. We investigated the distribution of monocytes in the bone marrow and in the peritoneal cavity as the primary site of infection. A large number of inflammatory Ly6C^hi^ monocytes infiltrated the peritoneal cavity between 6 and 12 h after CLP and dropped thereafter (Figures 5B,C). In parallel, the number of Ly6C^hi^ monocytes in the bone marrow strongly declined (Figures 5B,C). The majority of the remaining Ly6C^hi^ monocytes expressed CCR2, the receptor for CCL2 (Figure S3 in Supplementary Material). Within 96 h after CLP, the population of Ly6C^hi^ monocytes in the bone marrow was restored to homeostasis (Figure 5C). The egress of Ly6C^hi^ monocytes from bone marrow after CLP was preceded by the release of CCL2 in the peritoneal cavity (Figure 5D).

To investigate a potential association between monocytes and the expansion of the CD4^+^ DC population in the bone marrow, mice were treated with RS102895, a specific small molecule antagonist for CCR2 (48), before induction of sepsis. The administration of the CCR2 antagonist completely blocked the accumulation of CD4^+^ DCs in the bone marrow after CLP but did not significantly affect this population in sham mice (Figure 5E). The number of Ly6C^hi^ monocytes in the bone marrow of sham mice tended to be increased as a consequence of CCR2 inhibition (Figure 5F). In contrast, the application of the CCR2 antagonist did not affect the egress of monocytes from bone marrow after CLP (Figure 5F). Thus, the accumulation of CD4^+^ DCs in the bone marrow during sepsis is regulated by CCR2 but seems to be independent of monocyte mobilization.

### The Enlarged Population of CD4^+^ DCs after CLP Consists of Activated pDCs

**Figure 6:**
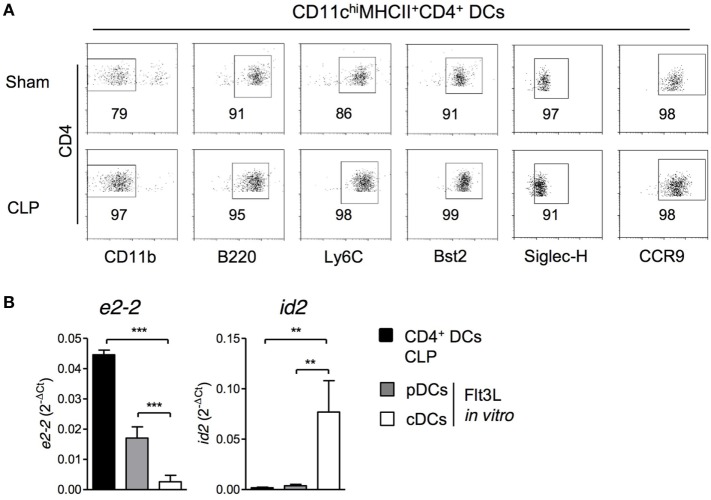
CD11c^hi^MHCII^+^CD4^+^ DCs express markers of pDCs. 36 h after sham or cecal ligation and puncture (CLP) operation, bone marrow cells (BMCs) were isolated. **(A)** Expression of CD11b, B220, Ly6C, Bst2, CCR9, and Siglec-H on gated CD11c^hi^MHCII^+^CD4^+^ DCs. Representative dot plots are shown. **(B)** CD11c^hi^MHCII^+^CD4^+^ DCs from BMC of CLP mice were sorted and the expression of *e2-2* and *id2* mRNA was determined by real-time PCR (*n* = 3; one replicate consisted of pooled cells from four mice). As control, BMC from individual sham mice (*n* = 3) were cultured in the presence of Flt3L and pDCs and conventional DCs were sorted as mentioned in the Section “Materials and Methods.” Bars indicate the mean + SD. Statistical differences were tested using one-way ANOVA followed by Newman–Keuls Multiple Comparison test. **p < 0.01; ***p < 0.001. DCs, dendritic cells; pDCs, plasmacytoid cells; cDC, conventional DCs.

After excluding a link between monocytes and CD4^+^ DCs in the bone marrow after CLP, we performed additional analyses of surface molecule expression. The CD4^+^ DCs in the bone marrow of septic mice did not express the myeloid marker CD11b but were positive for B220, Ly6C, Bst2, CCR9, and Siglec-H indicating that these cells represented pDCs (Figure 6A). In line with this assumption, sorted CD4^+^ DCs from septic mice expressed the transcription factor *e2-2* that is characteristic for pDCs but not *id2* that is expressed by conventional DCs (Figure 6B). In sham mice, the population of CD4^+^ DCs largely consisted of pDCs but additionally contained a small population of CD11b^+^ cells, most likely, conventional DCs (Figure 6A).

**Figure 7:**
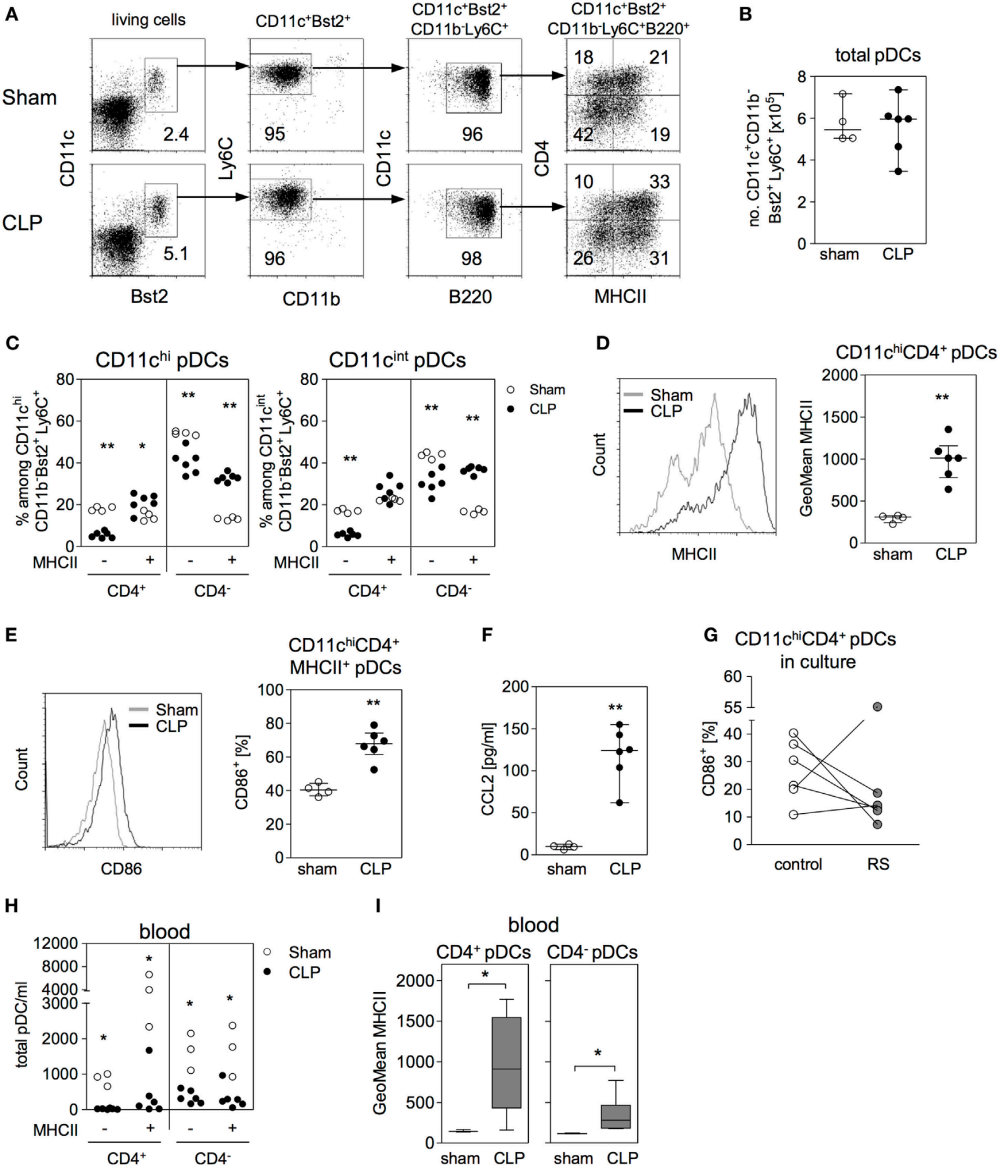
Identification of CD4^+^ DCs as activated plasmacytoid DCs (pDCs). **(A)** Gating strategy of pDCs. Bone marrow cells (BMC) were stained for CD11c, Bst2, CD11b, Ly6C, B220, major histocompatibility complex (MHC) class II, and CD4. pDCs were gated among living SSC^lo^/FSC^lo^ cells (equivalent to Figure 1B) as CD11c^+^Bst2^+^CD11b^−^Ly6C^+^B220^+^ and their expression of MHC class II and CD4 was analyzed. Dot plots are representative for one sham and one cecal ligation and puncture (CLP) mouse (36 h after surgery). Numbers indicate the percentage of gated cells or cells within the respective quadrant. **(B–I)** 16 h after surgery BMC, bone marrow lavage, and blood were analyzed. **(B)** Absolute number of CD11c^+^Bst2^+^CD11b^−^Ly6C^+^ pDCs in the bone marrow. **(C)** Expression of CD4 and MHC class II among the CD11c^hi^ pDCs and the CD11c^int^ pDCs (the regions for CD11c^hi^ and CD11c^int^ cells were set as shown in Figure 1B). **(D)** Geometric mean (GeoMean) of MHC class II expression on CD11c^hi^MHCII^+^CD4^+^ pDCs. **(E)** Frequency of CD86^+^ cells among CD11c^hi^MHCII^+^CD4^+^ pDCs. **(F)** Content of CCL2 in the bone marrow lavage. Data show median/interquartile range of individual mice (*n* = 4–6 per group). **(G)** BMCs from CLP mice were cultured for 24 h in the presence or absence of the CCR2 antagonist RS102895 (RS). Data show the percentage of CD11c^hi^CD4^+^ pDCs expressing CD86 for individual mice (*n* = 6 per group). **(H)** Number of pDC subpopulations in the blood of sham and CLP mice that were distinguished according to their expression of MHC class II and CD4 (*n* = 3–6 per group). **(I)** GeoMean of MHC class II on MHCII^+^CD4^+^ and MHCII^+^CD4^−^ pDCs in the blood of sham and CLP mice. Data show the median (interquartile range/range) of *n* = 3–6 mice per group. Statistical differences between sham and CLP were tested using the Mann–Whitney *U*-test. *p < 0.05; **p < 0.01; DCs, dendritic cells.

Recently, it has been reported that according to the expression of CD4 two maturation stages of pDCs in the bone marrow exist: CD4^−^ pDCs may develop into CD4^+^ pDCs and both populations may be released into the circulation (49). To get further insight into the expansion of CD11c^hi^CD4^+^ pDCs (that we had previously termed “CD4^+^ DCs”) in the bone marrow during sepsis, we investigated the expression of CD4 and MHC class II on CD11c^hi^ and CD11c^int^ pDCs among total CD11c^+^Bst2^+^Ly6C^+^B220^+^ pDCs (for gating, see Figure 7A). The discrimination between CD11c^hi^ and CD11c^int^ was performed equivalent to Figure 1B. 16 and 36 h after CLP, the number of total pDCs in the bone marrow did not differ between sham and CLP mice (Figure 7B; Figure S4A in Supplementary Material). Among the small subpopulation of CD11c^hi^ pDCs, we observed an increased percentage of MHCII^+^ pDCs at the expense of MHCII^-^ pDCs, both for the CD4^−^ and CD4^+^ subset at the earlier time point (Figure 7C). Moreover, the MHCII^+^CD4^+^ pDC from septic mice, expressed higher levels of MHC class II per cell and a larger part expressed CD86 in comparison with sham mice indicating an activated phenotype (Figures 7D,E). The activation of CD11c^hi^CD4^+^ pDCs was still apparent by 36 h after CLP but was not associated with *ifn*-α expression (Figures S4B–D in Supplementary Material). Among the major population of CD11c^int^ pDCs, a similar shift toward an increased percentage of MHC class II^+^ pDC was detected in the CD4^−^ but not in the CD4^+^ subpopulation (Figure 7C). Later on, by 36 h after CLP, the previously emerged shift toward MHCII^+^ pDCs was still apparent and additionally had established for CD11c^int^CD4^+^ pDC (Figure S4B in Supplementary Material).

Substantial amounts of CCL2 were present in the bone marrow by 16 h after CLP (Figure 7F). Considering that blocking of CCR2 prevented the expansion of the CD4^+^ DC population in the bone marrow after CLP *in vivo* and that pDCs are known to express CCR2 (50), we asked whether there exist a direct effect of the CCR2 antagonist on pDCs in the bone marrow. To address this question, BMCs were isolated 16 h after CLP and were cultured in the absence or presence of the CCR2 antagonist RS102895 before analysis of CD86 expression. The inhibition of CCR2 led to a decreased expression of CD86 on CD11c^hi^CD4^+^ pDCs (Figure 7G).

The number of pDCs that circulated in the blood of septic mice was strongly reduced irrespective of the subset (Figure 7H and Figure S4E in Supplementary Material for 16 and 36 h after surgery, respectively). Similar to pDCs in the bone marrow, circulating pDCs, especially the CD4^+^ subset, expressed higher levels of MHC class II after CLP (Figure 7I and Figure S4F in Supplementary Material for 16 and 36 h after surgery, respectively). Thus, the previously identified CD4^+^ DCs that expand in the bone marrow during sepsis are activated pDCs.

### Impact of pDCs on the Function of Splenic DCs *In Vivo*

**Figure 8:**
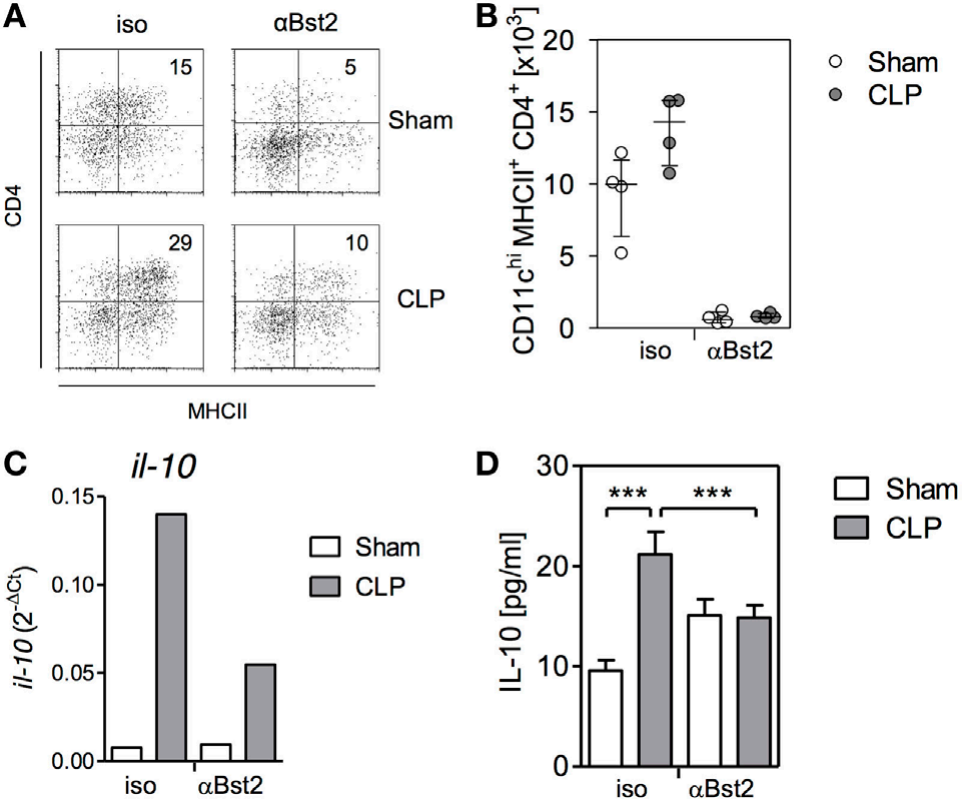
Depletion of plasmacytoid DCs reverses the bias of splenic dendritic cells (DCs) toward increased IL-10 production after cecal ligation and puncture (CLP) *in vivo*. Mice received antibodies against Bst2 or isotype control antibodies before and after sham surgery or CLP. **(A,B)** 36 hours after surgery, the number of CD11c^hi^MHCII^+^CD4^+^ DCs per mouse in the bone marrow was determined. **(A)** Representative dot plots of CD4 and MHCII expression on gated CD11c^hi^ cells. **(B)** Bar graph indicating the median/interquartile range of individual numbers of CD11c^hi^MHCII^+^CD4^+^ DCs (*n* = 4 mice/group). **(C)** Four days after surgery, splenic DCs were isolated, pooled per group (*n* = 4 mice/group), and the expression of *il-10* mRNA was determined by qPCR. **(D)** Four days after surgery, splenic DCs were isolated, pooled per group (*n* = 4 mice/group), and were stimulated with CpG *in vitro*. The content of IL-10 in the supernatant was quantified. Unstimulated cells did not release detectable amounts of IL-10 (not shown). Data show mean/SD of 3–5 replicates. Statistical differences were tested using one-way ANOVA followed by Bonferroni Multiple Comparison test. ***p < 0.001.

We have previously shown that 4 days after induction of sepsis, splenic DCs still maintain their bias toward increased IL-10 synthesis. At that time point, a large proportion of splenic DCs has been replaced by *de novo* generated DCs (37). Considering that pDCs in the bone marrow modulate the differentiation of DCs *in vitro* as shown above, we asked whether pDC influence the function of DCs *in vivo*. To address this issue, pDCs were depleted before CLP or sham surgery and splenic DCs were analyzed 4 days later. The population of the CD4^+^ pDCs was almost completely removed from bone marrow after application of the specific antibodies in sham and CLP mice (Figures 8A,B). Splenic DCs from CLP mice expressed clearly increased levels of *il-10* mRNA *ex vivo* and released more IL-10 after stimulation with CpG than DCs from sham mice when they had received the isotype control antibodies (Figures 8C,D). In contrast, prior depletion of pDCs reduced *ex vivo il-10* transcripts and CpG-induced IL-10 production in splenic DCs from septic mice but not in DCs from sham mice (Figures 8C,D). Thus, pDC favor the generation of IL-10-producing splenic DCs during sepsis.

## Discussion

In the present study, we investigated the so far unknown mechanisms that underlie the differentiation of dysfunctional DCs in the bone marrow during polymicrobial sepsis that are known to enhance the susceptibility to secondary infections. The common view is that the function of DCs is dependent on the local cytokine milieu in the tissue where the DCs reside (51). Moreover, environmental signals in the bone marrow, such as PGE_2_ and IL-10, may influence the development of DCs from their precursor cells and thereby induce a long-lasting alteration of the function of finally differentiated DCs (52, 53). The cytokine milieu in the bone marrow is supported by mesenchymal stromal cells (54). In addition, circulating cytokines may reach the bone marrow. Here, we provide first evidence that activated CD11c^hi^MHCII^+^CD4^+^ pDCs may represent a check-point during the differentiation of DCs from progenitor cells in the bone marrow during systemic bacterial infection. These activated CD4^+^ pDCs accumulated in the bone marrow after CLP and were largely responsible for the functional reprogramming of differentiating BMDCs toward increased IL-10 production *in vitro*. Finally, depletion of pDCs reversed the dysfunctional phenotype of splenic DCs during sepsis *in vivo*. IL-10 is released by diverse leukocytes during sepsis and increases the susceptibility to secondary infections (55–59). It remains to be elucidated to which extend the release of IL-10 by DCs is involved in this process.

Dendritic cells are continuously replaced by *de novo* generated pre-DCs released from the bone marrow (10). In case of inflammation DCs may additionally differentiate from monocytes that exit the bone marrow and are recruited to peripheral tissues (19). In GM-CSF cultures, early DC progenitors and monocytes give rise to DCs (60). It remains unclear at which developmental step pDCs act on BMDC differentiation and favor the differentiation of IL-10-secreting BMDCs. We assume that the modulation of DC precursor cells in the bone marrow by activated pDCs results in a sustained change of DC function in the periphery. This assumption is supported by the previous finding that the dysfunctional phenotype of splenic DCs after CLP is maintained even after resolution of the infection (29). Spaulding et al. have recently reported that activated, type I interferon-producing pDCs are present in the bone marrow during infection with *Plasmodium yoelii* (61), but they did not investigate the potential impact of these pDCs on the differentiation of other immune cells. We did not find evidence for IFN-α production by CD4^+^ pDCs in the bone marrow of septic mice, at least by 36 h after CLP. Thus, it is unlikely that IFN-α is responsible for the pDC-mediated shift of BMDC differentiation toward increased IL-10 production at that time point. Nevertheless, we cannot exclude that pDCs secreted type I IFN earlier during sepsis. Thus, unraveling the mechanisms that cause the expansion of CD11c^hi^CD4^+^ pDCs in the bone marrow during sepsis might help to find novel strategies to restrict the development of immunosuppression.

In line with a recent study (49), we found only few cycling CD11c^hi^CD4^+^ pDCs (according to their expression of Ki-67) in the bone marrow of sham and CLP mice. This finding suggests that the expansion of this population was not caused by enhanced proliferation. The rise in the number of CD11c^hi^CD4^+^ pDCs in the bone marrow during sepsis was dependent on signaling through MyD88, but not on TLR4. We assume that lipopolysaccharides from *Escherichia coli*, the major gut commensal, and TLR4 agonist, did not play a role in the activation of CD4^+^ pDCs in the bone marrow after CLP. Moreover, this finding might explain why the administration of lipopolysaccharide that is frequently used to mimic sepsis does not induce the differentiation of dysfunctional DCs *in vivo* and thus does not fully reflect sepsis-induced immunomodulation (62). The MyD88-associated receptor that confers the activation of pDCs during CLP-induced sepsis is currently under investigation.

Interestingly, we observed that the expansion of CD11c^hi^CD4^+^ pDCs was entirely controlled by CCR2 and S1P receptors, both well-known molecules with a crucial role in cell migration (63). This observation initially prompted us to assume that CD11c^hi^CD4^+^ pDCs themselves migrated into the bone marrow during sepsis. Strother et al. have recently reported that polymicrobial sepsis causes a loss of pDCs in the spleen (32) for unknown reasons. Since the population of CD4^+^ pDCs in the bone marrow was not reduced in splenectomized mice during sepsis, we can exclude the migration of pDCs from the spleen into the bone marrow. Moreover, we did not find an increased number of pDCs in the blood that would support the assumption of pDC migration into the bone marrow. Rather, the number of circulating pDCs of either subtype was strongly reduced possibly due to the recruitment into inflamed tissues (64). The few pDCs in the blood displayed an activated phenotype similar to their counterparts in the bone marrow (according to their increased expression of MHC class II) indicating prior stimulation at so far unknown sites.

Alternatively, we considered the indirect role of other migrating immune cells such as T lymphocytes or monocytes in the expansion of the CD11c^hi^CD4^+^ pDCs population in the bone marrow. A hallmark of sepsis is the profound depletion of T cells in secondary lymphoid and non-lymphoid tissues and in the blood that usually takes place within 24 h after induction of sepsis due to apoptosis (41, 42). In contrast, we observed a so far unrecognized expansion of CD4^+^ T cells in the bone marrow that paralleled the rise in the CD11c^hi^CD4^+^ pDC number and likewise could be inhibited by the administration of FTY720. Despite the similar behavior of CD4^+^ T cells and CD4^+^ pDCs in the bone marrow during sepsis, we did not find a causal relationship between these two populations as the size of the activated CD11c^hi^CD4^+^ pDC population was not affected in T cell-deficient mice. Since FTY720 is a functional inhibitor of multiple S1P receptors, further studies using selective receptor antagonists are required to clarify whether the expansion of the CD4^+^ T cells and CD4^+^ pDCs in the bone marrow during sepsis occurs through the same S1P signaling pathway. S1P signaling is also involved in the maintenance of vascular stability, survival, cytokine secretion, and other biological processes (65) that might indirectly cause the accumulation of these cells in the bone marrow.

As expected, Ly6C^hi^ inflammatory monocytes in the bone marrow highly expressed CCR2. Ligands of CCR2 induce the egress of monocytes from the bone marrow (66). Surprisingly, the application of a CCR2 antagonist prevented the rise in the CD11c^hi^CD4^+^ pDC number in the bone marrow but did not affect the egress of monocytes. The latter finding is in contrast to previous reports that show that blocking of CCR2 causes the retention of monocytes in the bone marrow during infection with *Listeria monocytogenes* or after application of a TLR4 agonist (18, 67). A recent study shows that CX3CR1 plays a major, possibly more important, role in the mobilization of monocytes during polymicrobial sepsis (68). Nevertheless, the question arose why blocking of CCR2 impaired the accumulation of CD11c^hi^CD4^+^ pDCs independent of monocytes. pDCs express CCR2 that mediates their release from bone marrow and migration into the periphery (18, 50). Thus, one might expect that blocking of CCR2 rather increases the number of pDCs in the bone marrow that is in contrast to our findings. Although RS102895 is a highly specific CCR2 antagonist (48), we cannot finally exclude any so far unknown side effects that may prevent pDC accumulation in the bone marrow in a CCR2-independent manner. Additional studies using alternative inhibitors, antibodies against CCR2, or CCR2-deficient mice are necessary to confirm the RS102895-mediated effect. Nevertheless, we considered an alternative role of CCR2 that is distinct to cellular trafficking.

We found an additional function of CCR2 when we took a closer look on the distribution of all pDCs on the distinct subpopulations defined by their expression of CD4 and MHC class II. While the number of CD11c^hi^CD4^+^ pDCs increased in the bone marrow after CLP, the number of total pDCs did not change. We observed a redistribution of CD11c^hi^ pDCs from MHC class II negative to positive, both, in the CD4^+^ and in the CD4^−^ pDC subpopulation. A similar pattern was found for CD11c^int^ pDCs though it appeared slightly delayed in the CD4^+^ pDCs subtype. It appears that the septic insult stimulated pDCs in the bone marrow for enhanced expression of MHC class II, CD86, and CD11c that finally increased the absolute number of CD11c^hi^MHCII^+^CD4^+^ pDCs. Therefore, we suggest that the expansion of the CD11c^hi^CD4^+^ pDC population reflected the activation of already existing pDCs in the bone marrow during sepsis. With regard to the function of CCR2 in this process, we provide first evidence that CCR2 contributed to the activation of CD4^+^ pDCs as the expression of CD86 on CD4^+^ pDCs in *ex vivo* cultured BMC was reduced in the presence of a CCR2 inhibitor. CCL2, the major ligand of CCR2 was not only found in the peritoneal cavity but also in the bone marrow where it might directly act on pDCs. Transferred to our *in vivo* study, we speculate that the administration of the CCR2 inhibitor prevented the activation of CD4^+^ pDCs in the bone marrow that subsequently did not acquire the CD11c^hi^ phenotype. Our hypothesis is strengthened by a previous report that shows that CCR2 promotes the maturation of conventional DCs and Langerhans cells (69). A similar role in the maturation of conventional DCs has been ascribed to S1P (70). Further investigation is required to examine whether S1P and CCR2 ligands cooperate in the activation of pDCs during polymicrobial sepsis and if so, which signaling pathways are involved. Since blocking of CCR2 has already proven protective in a severe sepsis model (71), it might be a promising candidate to prevent sepsis-induced immunosuppression.

Askenase et al. have recently shown that resident, mature NK cells in the bone marrow receive signals derived from infected tissues in the periphery and subsequently modulate the function of differentiating monocytes before these are released into the circulation (72). We propose the more general hypothesis that pDCs and NK cells and probably other leukocytes do not only act as effector cells in the periphery but moreover induce a functional program in differentiating myeloid cells in the bone marrow and thereby shape the immune response during infection.

## Ethics Statement

All animal experiments were performed according to the ethical principles and guidelines for scientific experiments. The protocol has been approved by the local ethic committee Landesamt für Natur-, Umwelt-, und Verbraucherschutz (LANUV), North-Rhine-Westphalia.

## Author Contributions

AS designed and performed experiments, analyzed the data, and wrote the paper. SP, SS, SA, RM-N, WH designed and performed experiments and analyzed the data. MN, MB, MG, A-CA performed experiments. FW and EP contributed to FACS analyses. AW provided support in cell sorting. MD and WH contributed materials and animals. SF designed the study and wrote the paper. All authors reviewed and approved the final version of the manuscript.

## Conflict of Interest Statement

The authors declare that the research was conducted in the absence of any commercial or financial relationships that could be construed as a potential conflict of interest.
