# Supplementary material for: Sphingosine 1-Phosphate- and C-C Chemokine Receptor 2-Dependent Activation of CD4+ Plasmacytoid Dendritic Cells in the Bone Marrow Contributes to Signs of Sepsis-Induced Immunosuppression
Source: Front Immunol. 2017 Nov 23;8:1622. doi: 10.3389/fimmu.2017.01622 (PMC5703700; doi:10.3389/fimmu.2017.01622)
Supplement: Supplementary file 1 [file data_sheet_1.pdf]

Supplementary Material

Sphingosine 1-phosphate- and CCR2-dependent activation of CD4$^+$ plasmacytoid dendritic cells in the bone marrow contributes to signs of sepsis-induced immunosuppression

Anna Smirnov, Stephanie Pohlmann, Melanie Nehring, Stefanie Scheu, Shafaqat Ali, Ritu Mann-Nüttel, Anne-Charlotte Antoni, Wiebke Hansen, Manuela Buettner, Miriam J. Gardiasch, Astrid M. Westendorf, Florian Wirsdörfer, Eva Pastille, Marcel Dudda, Stefanie B. Flohé*

*** Correspondence:** Stefanie B. Flohé, stefanie.flohe@uk-essen.de

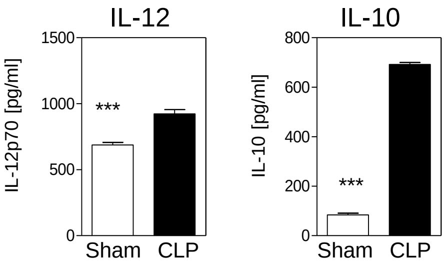

Supplementary Figure 1. Cytokine secretion of BMDC. Thirty-six h after sham or CLP operation, bone marrow cells were isolated and BMDC were generated for 9 d *in vitro* using granulocyte-macrophage colony-stimulating factor. BMDC were stimulated with CpG and the content of IL-12 and IL-10 in the supernatants was determined. Unstimulated BMDCs did not secrete detectable amounts of IL-12 or IL-10 (data not shown). Data show mean+SD of triplicate cultures from one representative out of five experiments. Statistical differences were tested using Student t-test. ***, p<0.001. BMDC, bone marrow-derived dendritic cells
