# Supplementary material for: Sphingosine 1-Phosphate- and C-C Chemokine Receptor 2-Dependent Activation of CD4+ Plasmacytoid Dendritic Cells in the Bone Marrow Contributes to Signs of Sepsis-Induced Immunosuppression
Source: Front Immunol. 2017 Nov 23;8:1622. doi: 10.3389/fimmu.2017.01622 (PMC5703700; doi:10.3389/fimmu.2017.01622)
Supplement: Supplementary file 2 [file data_sheet_2.pdf]

Supplementary Material

Sphingosine 1-phosphate- and CCR2-dependent activation of $CD4^+$ plasmacytoid dendritic cells in the bone marrow contributes to signs of sepsis-induced immunosuppression

Anna Smirnov, Stephanie Pohlmann, Melanie Nehring, Stefanie Scheu, Shafaqat Ali, Ritu Mann-Nüttel, Anne-Charlotte Antoni, Wiebke Hansen, Manuela Buettner, Miriam J. Gardiasch, Astrid M. Westendorf, Florian Wirsdörfer, Eva Pastille, Marcel Dudda, Stefanie B. Flohé*

*** Correspondence:** Stefanie B. Flohé, stefanie.flohe@uk-essen.de

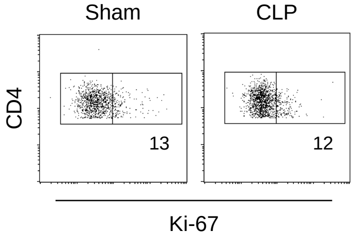

Supplementary Figure 2. Expression of Ki-67 on $CD4^+$ DCs in the bone marrow. Thirty-six h after sham or CLP operation, bone marrow cells were isolated. After staining for CD11c, MHC class II, and CD4 (as described in the section Materials and Methods) the cells were incubated in Fixation/Permeabilization solution (Foxp3 Staining Buffer Set, eBioscience) for 2 h at 4°C. After washing with Permeabilization Buffer, the cells were stained with a fluorochrome-labeled anti-Ki-67 antibody (clone SolA15; eBioscience) for 15 min in the dark at 4°C, and finally washed with Permeabilization Buffer. The dot plots show the expression of Ki-67 in gated $CD11c^{hi}MHCII^+CD4^+$ DCs from one representative sham and one CLP mouse. The numbers indicate the percentage of positive cells among gated cells. DCs, dendritic cells
