# Supplementary material for: Sphingosine 1-Phosphate- and C-C Chemokine Receptor 2-Dependent Activation of CD4+ Plasmacytoid Dendritic Cells in the Bone Marrow Contributes to Signs of Sepsis-Induced Immunosuppression
Source: Front Immunol. 2017 Nov 23;8:1622. doi: 10.3389/fimmu.2017.01622 (PMC5703700; doi:10.3389/fimmu.2017.01622)
Supplement: Supplementary file 3 [file data_sheet_3.pdf]

Supplementary Material

Sphingosine 1-phosphate- and CCR2-dependent activation of $CD4^+$ plasmacytoid dendritic cells in the bone marrow contributes to signs of sepsis-induced immunosuppression

Anna Smirnov, Stephanie Pohlmann, Melanie Nehring, Stefanie Scheu, Shafaqat Ali, Ritu Mann-Nüttel, Anne-Charlotte Antoni, Wiebke Hansen, Manuela Buettner, Miriam J. Gardiasch, Astrid M. Westendorf, Florian Wirsdörfer, Eva Pastille, Marcel Dudda, Stefanie B. Flohé*

*** Correspondence:** Stefanie B. Flohé, stefanie.flohe@uk-essen.de

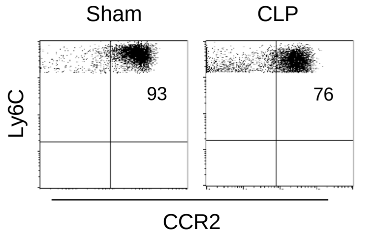

Supplementary Figure 3. Expression of CCR2 on monocytes in the bone marrow. Thirty-six h after sham or CLP operation, bone marrow cells were isolated. The cells were stained for CD11c, Ly6C, and CCR2 (clone 475301, eBioscience). The dot plots show the expression of CCR2 on gated $CD11c^-Ly6C^{hi}$ monocytes from one representative sham and one CLP mouse. The numbers indicate the percentage of positive cells among gated cells.
