# Supplementary material for: Sphingosine 1-Phosphate- and C-C Chemokine Receptor 2-Dependent Activation of CD4+ Plasmacytoid Dendritic Cells in the Bone Marrow Contributes to Signs of Sepsis-Induced Immunosuppression
Source: Front Immunol. 2017 Nov 23;8:1622. doi: 10.3389/fimmu.2017.01622 (PMC5703700; doi:10.3389/fimmu.2017.01622)
Supplement: Supplementary file 4 [file data_sheet_4.pdf]

Supplementary Material

Sphingosine 1-phosphate- and CCR2-dependent activation of CD4^{+} plasmacytoid dendritic cells in the bone marrow contributes to signs of sepsis-induced immunosuppression

Anna Smirnov, Stephanie Pohlmann, Melanie Nehring, Stefanie Scheu, Shafaqat Ali, Ritu Mann-Nüttel, Anne-Charlotte Antoni, Wiebke Hansen, Manuela Buettner, Miriam J. Gardiasch, Astrid M. Westendorf, Florian Wirsdörfer, Eva Pastille, Marcel Dudda, Stefanie B. Flohé*

*** Correspondence:** Stefanie B. Flohé, stefanie.flohe@uk-essen.de

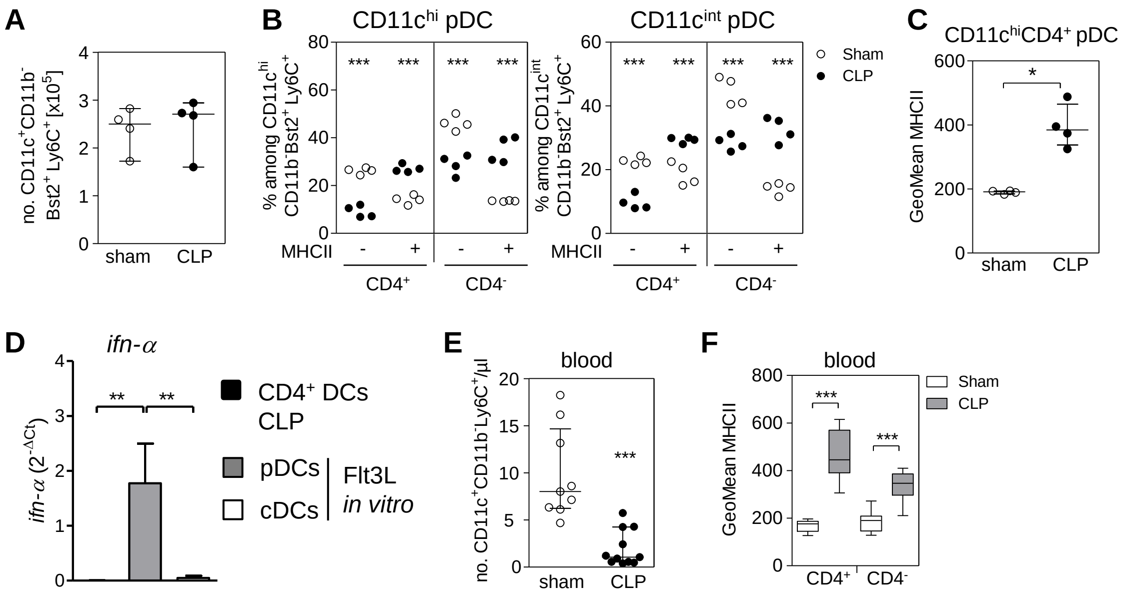

Supplementary Figure 4. Distribution of plasmacytoid DCs on diverse subpopulations according to their expression of CD4 and MHC class II. Thirty-six h after sham or CLP operation, bone marrow cells and blood leukocytes were isolated and stained for CD11c, MHC class II, CD4, CD11b, Bst2, and Ly6C (as described in the section Materials and Methods). (A) Absolut numbers of CD11c^{+}CD11b^{-}Bst2^{+}Ly6C^{+} pDCs in the bone marrow. (B) Frequency of MHC class II^{-} and MHC class II^{+} pDCs among CD4^{-} and CD4^{+} pDCs in the bone marrow. Separate analyses of CD11c^{hi} and CD11c^{int} pDCs from individual mice are shown. (C) Geometric mean (GeoMean) of MHC class II expression on CD11c^{hi}MHCII^{+}CD4^{+} pDC. Data show individual values and/or the median (interquartile range/range) of n=4 mice per group. (D) CD11c^{hi}CD4^{+}MHCII^{+} DCs were sorted from bone marrow of CLP mice (n=3; one replicate consisted of pooled cells from 4 mice). As control, pDCs and cDCs from individual Flt3L cultures of sham mice (n=3) were stimulated with 0.05 µM CpG 2216 complexed to DOTAP for 4 h. RNA was prepared and the expression of *ifn-α* mRNA was determined by real-time PCR. Statistical differences were tested using One-way ANOVA followed by Newman-Keuls Multiple Comparison test. (E) Absolute numbers of CD11c^{+}CD11b^{-}Ly6C^{+} pDCs in the blood. (F) GeoMean of MHC class II on MHCII^{+}CD4^{+} and MHCII^{+}CD4^{-} pDCs in the blood. Data show individual values and/or the median (interquartile range/range) of n=9-11 mice per group. Statistical differences were tested using the Mann-Whitney U-test. *, $p<0.05$ ***, $p<0.001$. DCs, dendritic cells; pDCs, plasmacytoid DCs; cDCs, conventional DCs
